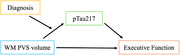# White matter perivascular spaces are associated with lower phosphorylated tau and preserved cognitive function in Alzheimer’s Disease

**DOI:** 10.1002/alz70861_108995

**Published:** 2025-12-23

**Authors:** Celine H.L. Huang, Si Won Ryoo, Sofia Perfetto, William Z. Lin, Myuri Ruthirakuhan, Yuen Yan Wong, Malcolm Binns, Stephen R. Arnott, Robert Bartha, Sean Symons, Julie Ottoy, Bradley J. MacIntosh, Maged Goubran, Jennifer S Rabin, Sandra E. Black, Joel Ramirez, Walter Swardfager

**Affiliations:** ^1^ University of Toronto, Toronto, ON Canada; ^2^ Sunnybrook Research Institute, Toronto, ON Canada; ^3^ Rotman Research Institute, Baycrest Academy for Research and Education, Toronto, ON Canada; ^4^ Dalla Lana School of Public Health, University of Toronto, Toronto, ON Canada; ^5^ Indoc Systems, Toronto, ON Canada; ^6^ Western University, London, ON Canada; ^7^ Robarts Research Institute, London, ON Canada; ^8^ Sunnybrook Health Sciences Centre, Toronto, ON Canada; ^9^ Dr. Sandra E. Black Centre for Brain Resilience and Recovery, Toronto, ON Canada; ^10^ University of Toronto Scarborough, Toronto, ON Canada; ^11^ Canadian Partnership for Stroke Recovery, Toronto, ON Canada; ^12^ Heart and Stroke Foundation Canadian Partnership for Stroke Recovery, Toronto, ON Canada; ^13^ Division of Neurology, Department of Medicine, University of Toronto, Toronto, ON Canada; ^14^ Dr. Sandra Black Centre for Brain Resilience & Recovery, Toronto, ON Canada; ^15^ Toronto Dementia Research Alliance, Toronto, ON Canada; ^16^ LC Campbell Cognitive Neurology Research Unit, Sunnybrook Research Institute, Toronto, ON Canada

## Abstract

**Background:**

Enlarged perivascular spaces (PVS) are features of small vessel cerebrovascular diseases (CVD) and have been implicated in Alzheimer’s disease (AD). Questions remain, however, on the role that PVS may play in the pathophysiology of AD. The current study focuses on phosphorylated tau217 (pTau217), a blood biomarker of AD, in relation to PVS among individuals with different cognitive statuses and neurodegenerative diseases.

**Method:**

This cross‐sectional study used data from the Ontario Neurodegenerative Disease Research Initiative. Participants were classified as AD or CVD based on their clinical diagnosis. T1‐weighted MRI scans were processed to obtain white matter (WM) PVS volumes, and plasma pTau217 levels were quantified using SIMOA. A composite score for executive function (EF) was calculated from the Trail Making Test B, Stroop colour‐word: interference and switching, Verbal letter and category Fluency Tests, and the Wechsler Abbreviated Scale of Intelligence: matrix reasoning. Linear regression models were used to examine the associations between WM PVS volumes, pTau217 and EF, while adjusting for age, sex, APOE4 status, and vascular risk factors (hypertension, BMI). Clinical diagnosis was included as an interaction term to examine condition‐specific differences. A moderated‐mediation analysis was conducted to examine whether the relationship between WM PVS, pTau217, and EF varied by clinical diagnosis.

**Result:**

80 participants were included in this study, 40 with AD (age=72±8,58%female), and 40 with CVD (age=72±7,58%female). The association between WM PVS volumes and pTau217 was moderated by clinical diagnosis (β=‐0.238, *p* =0.015), whereby higher WM PVS volumes predicted lower pTau217 levels in the AD group (β=‐0.170, *p* =0.033), but not the CVD group (β=0.018, *p* =0.763). Higher pTau217 levels also predicted lower EF overall (β=‐1.795, *p* =0.017). The indirect effect of WM PVS volume on EF via pTau217 was significant in the AD group (β=0.492, 95%CI [0.058,1.117]), but not in the CVD group (β=‐0.078, 95%CI [‐0.481,0.269]).

**Conclusion:**

These findings suggest that enlarged WM PVS are associated with lower peripheral pTau217 levels, which may contribute to preserved cognitive function in AD patients, but not in CVD patients. Understanding how PVS dynamics differ across neurodegenerative diseases could provide insight into their distinct origins and clinical consequences, guiding the development of targeted therapies.